# Supplementary material for: The Q163C/Q309C mutant of αMI-domain is an active variant suitable for NMR characterization
Source: PLoS One. 2023 Jan 25;18(1):e0280778. doi: 10.1371/journal.pone.0280778 (PMC9876370; doi:10.1371/journal.pone.0280778)
Supplement: S1 Fig — (DOCX) [file pone.0280778.s001.docx]

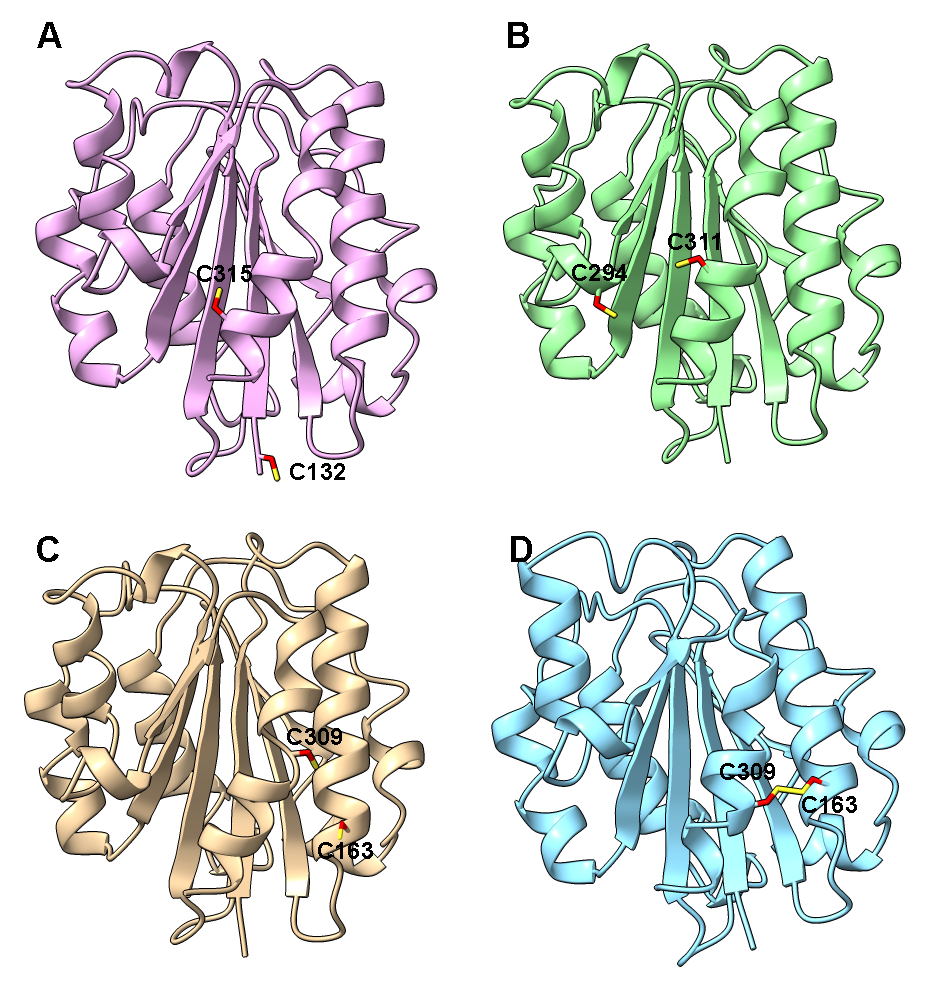


**Figure 1**. Models of the disulfide bond mutants. A) Model of the D132C/K315C mutant in the inactive form. B) Model of the D294C/Q311C mutant in the inactive form. C) Model of the Q163C/Q309C mutant in the inactive form. D) Model of the Q163C/Q309C mutant in the active form. The inactive model is based on the PDB structure 1JLM. The active model is based on the PDB structure 1IDO. The side chains of cysteines are shown in the stick representation.


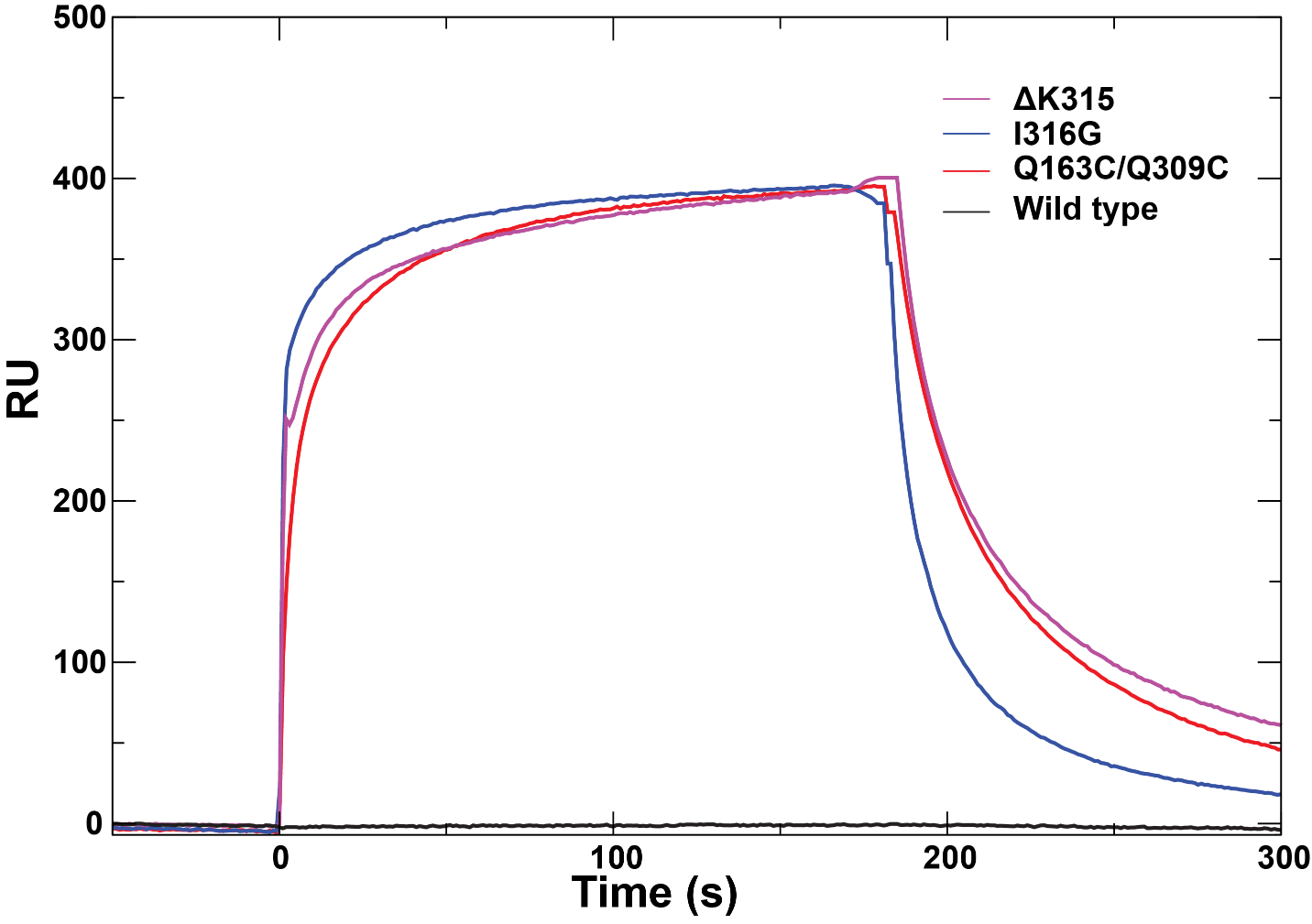


**Figure 2**. SPR response curves of a C3d-functionalized sensor treated with 3 µM of ΔK315 (magenta), I316G (blue), Q163C/Q309C (red), or wild type (black) α_M_I-domain. Injection of α_M_I-domain started at time zero and ended after 180 seconds.


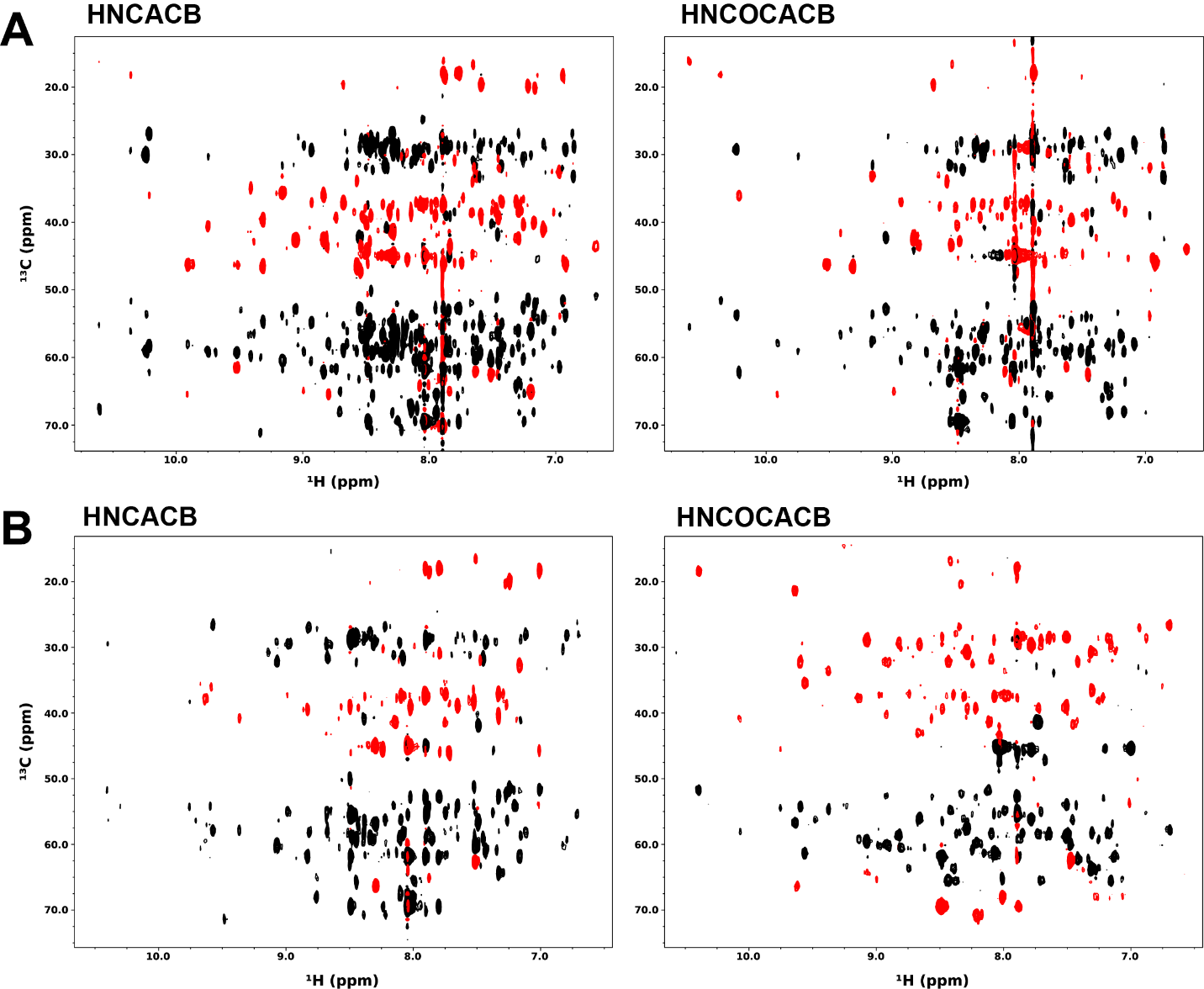


**Figure 3**. Projections of HNCACB and HNCOCACB for ^2^H,^15^N,^13^C-labeled Q163C/Q309C mutant bound to Mg^2+^ (A) and Co^2+^ (B). Positive signals are represented by black contours and negative signals are represented by red contours. Both samples also contained 10 mM glutamate.


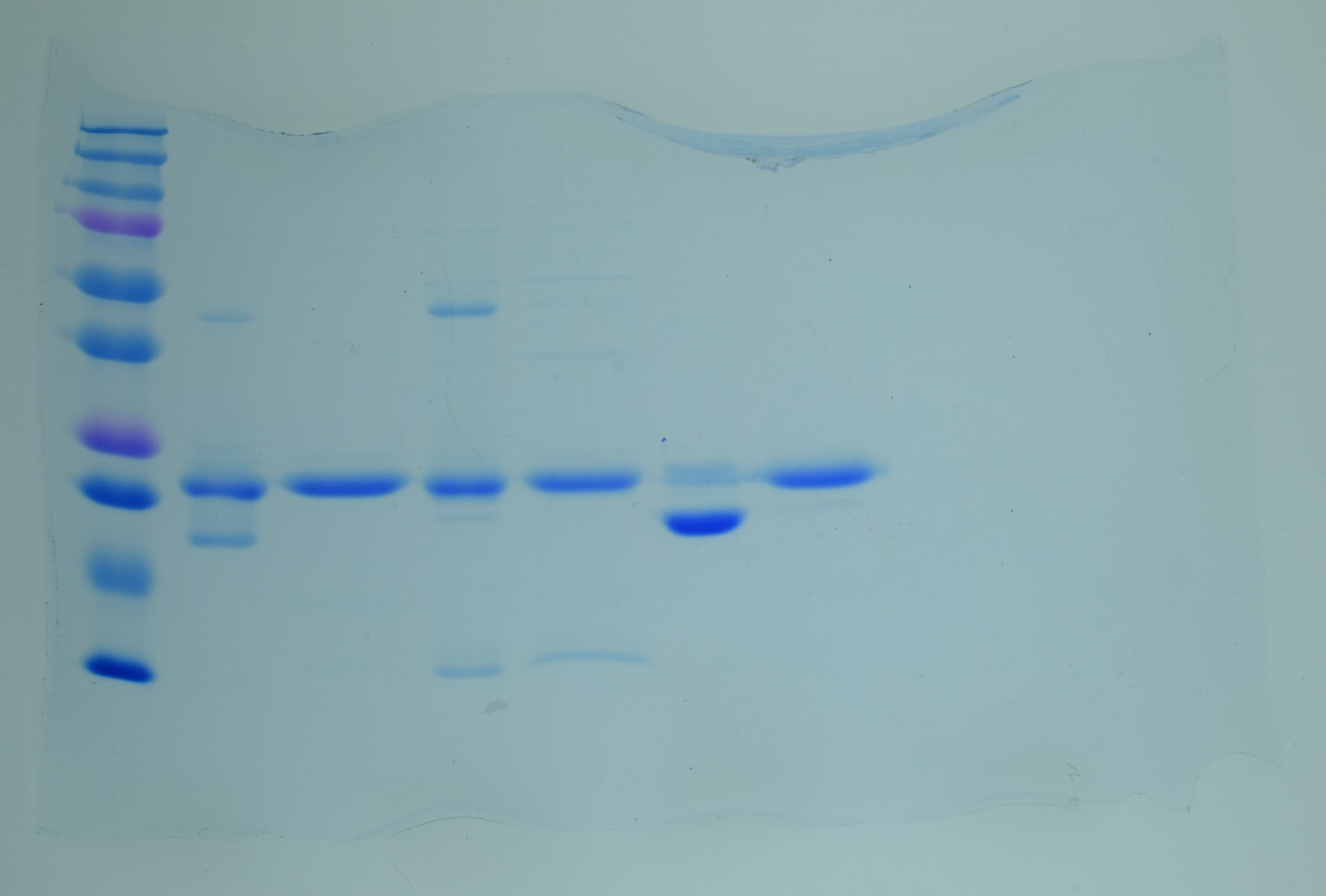


**Figure 4**. Unmodified and uncropped gel picture for Figure 1A.


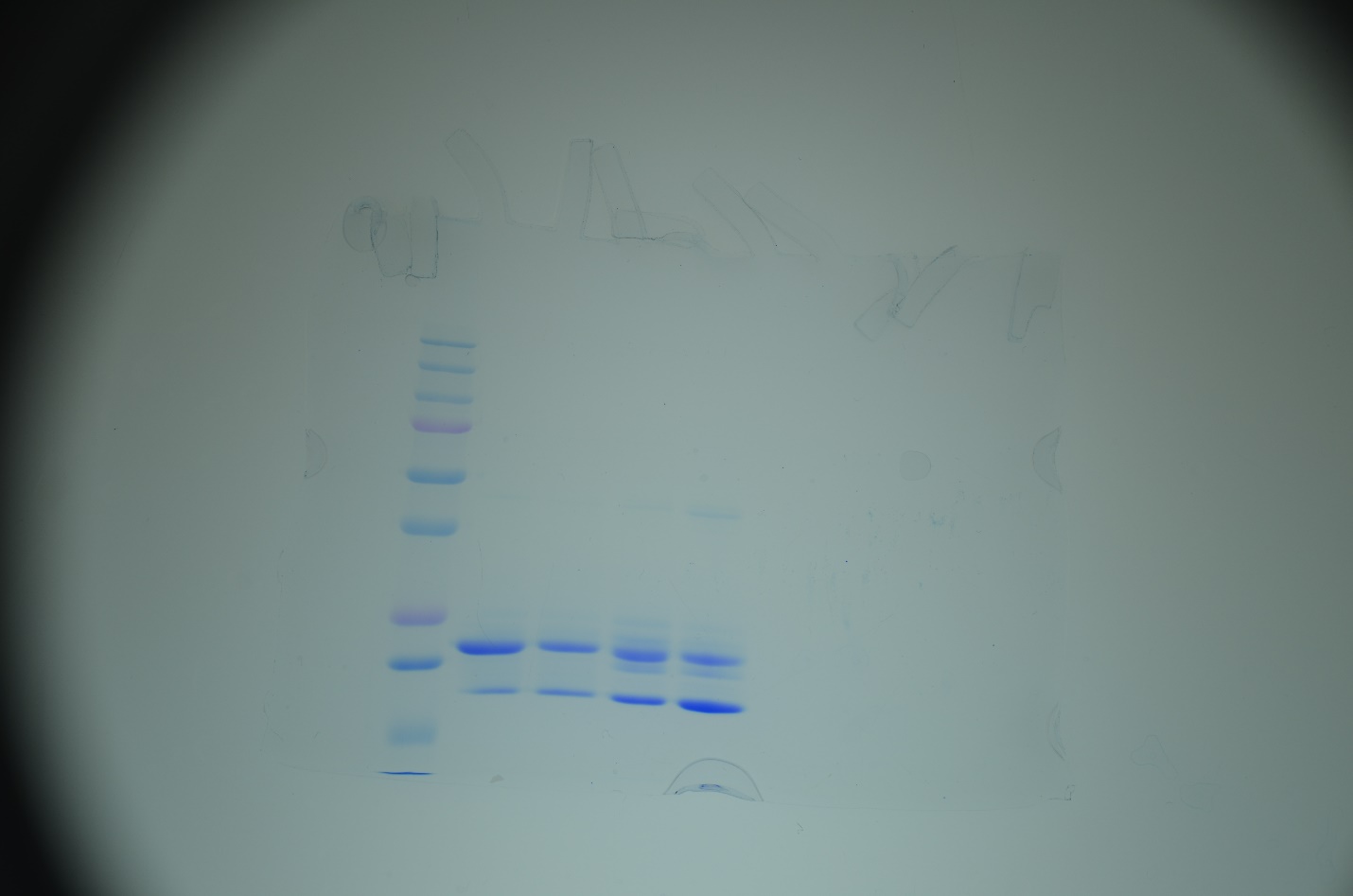


**Figure 5**. Uumodified and uncropped gel picture for Figure 1B.


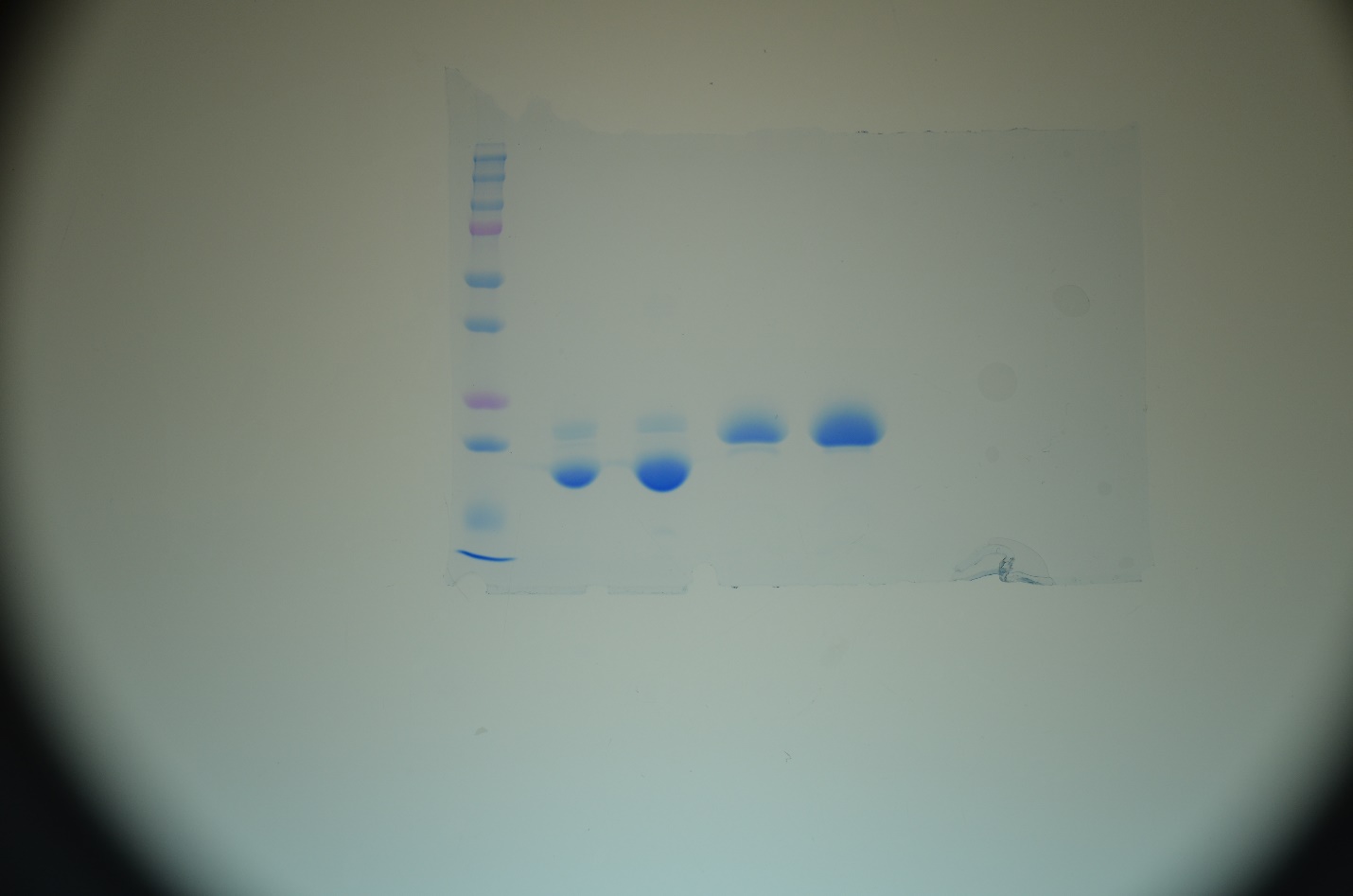


**Figure 6**. Unmodified and uncropped gel picture for Figure 1C. The last two lanes are the Q163C/Q309C mutant samples produced in BL21(DE3) and OrigamiB(DE3) in the presence of 10 mM DTT.


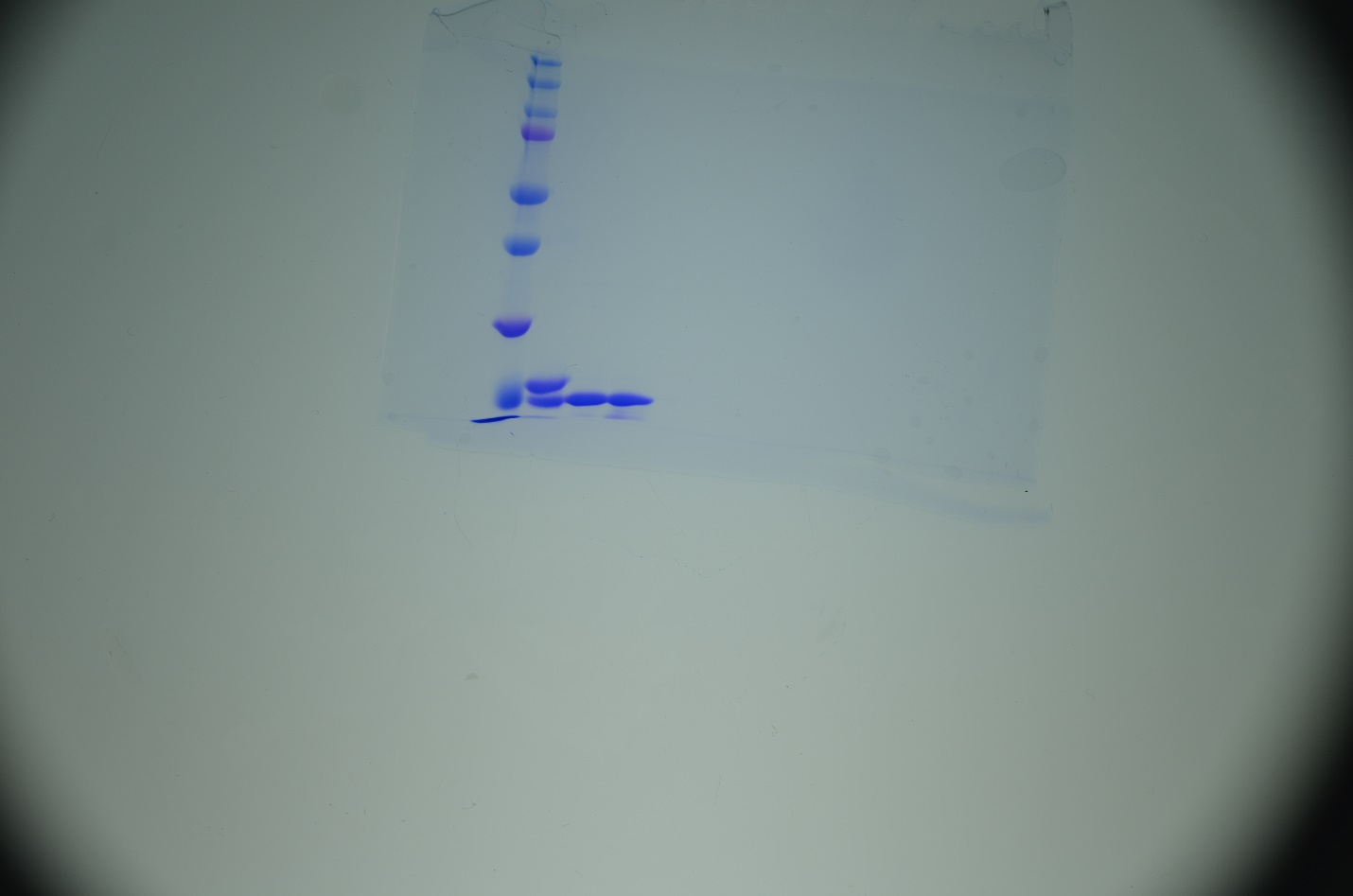


**Figure 7**. Unmodified and uncropped gel picture for Figure 6.
