# Supplementary material for: The Q163C/Q309C mutant of αMI-domain is an active variant suitable for NMR characterization
Source: PLoS One. 2023 Jan 25;18(1):e0280778. doi: 10.1371/journal.pone.0280778 (PMC9876370; doi:10.1371/journal.pone.0280778)
Supplement: S1 Table — (DOCX) [file pone.0280778.s002.docx]

| Residue Number | Amide Hydrogen Chemical Shifts (ppm) | | |
| --- | --- | --- | --- |
|  | Co^2+^ | Mg^2+^ | PCS |
| 150 | 5.74 | 8.31 | -2.57 |
| 151 | 6.76 | 8.05 | -1.29 |
| 152 | 6.68 | 7.6 | -0.92 |
| 153 | 7.52 | 8.35 | -0.83 |
| 154 | 6.79 | 7.27 | -0.48 |
| 155 | 7.09 | 7.42 | -0.33 |
| 160 | 8.15 | 8.22 | -0.07 |
| 171 | 5.55 | 5.49 | 0.06 |
| 172 | 8.02 | 7.89 | 0.13 |
| 173 | 7.36 | 7.14 | 0.22 |
| 185 | 9.48 | 9.33 | 0.15 |
| 188 | 8.10 | 8.01 | 0.09 |
| 189 | 8.82 | 8.73 | 0.09 |
| 190 | 8.48 | 8.41 | 0.07 |
| 203 | 8.39 | 8.22 | 0.17 |
| 204 | 8.21 | 8.46 | -0.25 |
| 213 | 9.25 | 10.61 | -1.36 |
| 214 | 8.02 | 8.54 | -0.52 |
| 215 | 7.67 | 8.01 | -0.34 |
| 227 | 8.01 | 7.95 | 0.06 |
| 229 | 6.99 | 6.94 | 0.05 |
| 251 | 8.11 | 9.06 | -0.95 |
| 253 | 9.56 | 10.21 | -0.65 |
| 258 | 7.15 | 7.34 | -0.19 |
| 259 | 7.49 | 7.65 | -0.16 |
| 260 | 8.41 | 8.53 | -0.12 |
| 261 | 8.28 | 8.38 | -0.10 |
| 262 | 7.65 | 7.71 | -0.06 |
| 275 | 9.63 | 8.681 | 0.95 |
| 276 | 9.14 | 8.1 | 1.04 |
| 277 | 8.29 | 7.2 | 1.09 |
| 278 | 9.63 | 8.99 | 0.64 |
| 280 | 8.91 | 7.64 | 1.27 |
| 281 | 9.07 | 8.11 | 0.96 |
| 283 | 8.33 | 7.9 | 0.43 |
| 288 | 7.25 | 7.16 | 0.09 |
| 296 | 7.14 | 6.97 | 0.17 |
| 298 | 8.67 | 8.47 | 0.20 |

Table. Co^2+^-induced Backbone amide hydrogen PCS of the Q163C/Q309C mutant used to calculate the magnetic susceptibility tensor.
