# Supplementary material for: The Q163C/Q309C mutant of αMI-domain is an active variant suitable for NMR characterization
Source: PLoS One. 2023 Jan 25;18(1):e0280778. doi: 10.1371/journal.pone.0280778 (PMC9876370; doi:10.1371/journal.pone.0280778)
Supplement: Attachment — Submitted filename: Response-v1.1.docx [file pone.0280778.s003.docx]

Reviewer #1: This manuscript is an elegant example of rational engineering to retain function, and enable structural/physical analysis. The goal is to study an ligand-binding domain of an integrin, in the active state. Previous work proposes mutations to "stabilize" the domain, but here it is shown that such mutations do not stabilize the domain. The authors propose new mutations that would lead to an active state whereby they would stabilize with an intramolecular disulphide. All the data presented supports the conclusion of a stable active domain, although it requires glutamate/Mg2+ to maintain a state suitable for NMR analysis. The paper is well written, figures are clear. The only issue is a description of the Co+-induced pseudocontact shifts are based on the assignments. I would like these assignments to be deposited in the BMRB or if you have state the accession number. I consider this important as the BMRB will assess the quality (accuracy) of assignment. I do agree the fitting of the PCS is convincing that all is good, but you need to deposit the data.

**Reply**: The chemical shifts of both the Mg^2+^ and Co^2+^ species of the Q163C/Q309C bound to glutamate have been deposited with BMRB. The accession numbers are 51714 for the Mg^2+^ species and 51716 for the Co^2+^ species. A detailed description of the assignment process is also included in the manuscript (lines 162 to 173). In particular, the Mg^2+^ species were assigned with HNCACB and HNCOCACB using conventional methods. To assign the Co^2+^ species, we used HNCACB and HNCOCACB of the Co^2+^ sample and the fact that PCS values are similar for amide hydrogen and nitrogen pairs if the atoms are not very close to the paramagnetic center to predict possible assignments. The proposed assignments were verified using CA and CB chemical shifts.

Reviewer #2: This very clearly written and presented MS makes a strong and logically argued case for the use of an engineered domain, from a key integrin (CR3), for ligand-binding and screening studies, and in particular for studies using NMR spectroscopy. The modified domain has two non-native cysteines that form a disulfide bond, presumably stabilising an active conformation of the domain that is able to bind ligands (unlike the wild type domain) via a MIDAS motif. Because of the many reported ligands for this domain and its well-established central role in immune responses, the topic is of quite wide interest.

It would be good to show (eg in supplementary) the position of the mutated residues and perhaps a predicted structure of the disulfide-containing molecule.

**Reply**: Models of the disulfide bond mutants have been added to the supporting information (Figure 1 in S1 figures).

Readers might be a little sceptical of how the authors were able to assign the amide nitrogens and protons for this mutant given the sub-optimal nature of the HSQC spectrum that contains many, well dispersed but very small peaks but also a region of more intense and overlapping peaks in the random-coil region. To reassure us, they should include in supplementary data a full account of this process including a method section and representative HNCACB and HNCOCACB spectra, and information regarding the extent of 15NH assignments (that should be deposited in a database) for both Mg and Co forms.

**Reply**: We apologize for neglecting to mention the details of our assignment process. In particular, we hope we have not given the false impression that all residues in the two metal-bound forms were assigned. Because our goal is to validate the structure, we only obtained enough assignments to allow the evaluation of the structure of the Q163C/Q309C mutant. As for the quality of the spectra, 3D spectra were collected using samples containing 1 mM divalent cation and 10 mM glutamate. These ligands significantly improved the spectral quality of the Q163C/Q309C mutant. This was mentioned in the methods section, and now we also added the detail to the results section. The samples were also deuterated to reduce relaxation and increase intensity. We have included projections of the HNCACB and HNCOCACB data in the supporting information (Figure 3 in S1 figure). Assignments have also been deposited with BMRB (accession code 51714 for the Mg^2+^ species and 51716 for the Co^2+^ species). A detailed description of the assignment process has been included in the methods section (lines 162 to 173). In total, 77 residues out of 186 non-proline residues in the Mg^2+^ form were assigned. Out of these 77 residues, 56 residues in the Co^2+^ form were assigned, 38 of which had HN PCS greater than 0.05 ppm.

Another improvement to this MS would be to include some KD values based on SPR studies using a dilution series of their domains (binding to immobilised C3d). The authors seem to have plenty of sample for the binding partners and access to the kit so it is puzzling why they have not already included such measurements. It looks like the alpha-helix truncation mutant might bind to C3d more tightly than the double-Q (disulfide) mutant since it has a slower off-rate. That does not really weaken their conclusions but is good to know.

**Reply**: We were unable to include full titration series for these active variants in our initial manuscript because non-specific interactions of active mutants with the sensor prevented the inclusion of data acquired with higher concentrations of the mutants. We have repeated these SPR experiments with lower concentrations of mutants and the data are now included in the manuscript. Kds obtained from equilibrium values of the responses at different concentrations showed the affinities of these mutants for C3d are similar and in agreement with reported values (lines 255-269, Figure 3). These data also confirmed what the reviewer already noted, that is the dissociation rate of the ΔK315 mutant is slower than the other mutants. However, we were not able to obtain good fittings of the association and dissociation phases to calculate the actual rates.

Small things:
Understanding.... is... (not are)
Data are... (not is)
And they seem to use the terms intracellular and intramolecular indiscriminately - I think they mean intermolecular (?)

**Reply**: We have carefully gone through the manuscript to correct all grammatical mistakes we can find. All mentions of “intracellular” have been replaced with “intramolecular”.
