# Supplementary material for: The Q163C/Q309C mutant of αMI-domain is an active variant suitable for NMR characterization
Source: PLoS One. 2023 Jan 25;18(1):e0280778. doi: 10.1371/journal.pone.0280778 (PMC9876370; doi:10.1371/journal.pone.0280778)
Supplement: Attachment — Submitted filename: Response2.docx [file pone.0280778.s004.docx]

Reviewer #1: The authors have addressed comments and questions of the review. The deposition of data to the BMRB is important.

**Response**: Thank you for your review.

Reviewer #2: The authors have addressed my concerns but please correct statement regarding SPR. You say 1 AU = 1 pg per mm. This should of course be 1 pg per mm2 (square mm)

**Response**: Thank you for noticing the mistake in the unit. We have changed it to “1 pg / mm^2^” (line 149).
